# The special considerations of gene therapy for mitochondrial diseases

**DOI:** 10.1038/s41525-020-0116-5

**Published:** 2020-03-02

**Authors:** Jesse Slone, Taosheng Huang

**Affiliations:** Division of Human Genetics, Cincinnati Children’s Hospital Medical Center, 3333 Burnet Avenue, Cincinnati, OH 45229 USA

**Keywords:** Diseases, Medical genetics, Genetics research

## Abstract

The recent success of gene therapy across multiple clinical trials has inspired a great deal of hope regarding the treatment of previously intractable genetic diseases. This optimism has been extended to the prospect of gene therapy for mitochondrial disorders, which are not only particularly severe but also difficult to treat. However, this hope must be tempered by the reality of the mitochondrial organelle, which possesses specific biological properties that complicate genetic manipulation. In this perspective, we will discuss some of these complicating factors, including the unique pathways used to express and import mitochondrial proteins. We will also present some ways in which these challenges can be overcome by genetic manipulation strategies tailored specifically for mitochondrial diseases.

## Introduction

Remarkable strides have been made in the field of gene therapy in recent years, and there is a growing sense in the field that the power of gene therapy and gene editing techniques such as CRISPR will soon allow for the treatment of a broad range of genetic disorders. Indeed, regulatory approval has recently been granted to gene replacement therapies for several disorders, including Leber congenital amaurosis type 2 (LCA2),^1^ spinal muscular atrophy type 1 (SMA1),^2^ and β-thalassemia.^3^ The latter result has the potential to be particularly impactful, as β-thalassemia is one of the most common inherited blood disorders in the world, affecting approximately 1 in 100,000 people globally.^4^ It is clear that gene replacement therapy is coming into its own, and for those interested in brushing up on the subject, the recent review by High and Roncarolo^5^ is an excellent starting point for a brief, but thorough, overview of the current state of the field.

Despite this remarkable progress, gene therapy for inherited mitochondrial disorders may present a unique and fascinating set of challenges that are not fully appreciated by those less acquainted with mitochondrial biology. Mitochondria are, of course, integral to the functioning of the cell, producing the bulk of the energy (in the form of ATP) needed by the cell through the process of oxidative phosphorylation (OXPHOS). The consequences of mutations in the mitochondrial genome (mtDNA) and mitochondria-related nuclear genes are often severe, and the prognosis of such a patient is usually quite poor. Thus, the value in being able to correct such genetic defects in patients is readily apparent. Gene therapy and CRISPR gene editing provide a great deal of promise in the field of medical genetics, but have certain limitations in the treatment of mitochondrial diseases that must be addressed if they are to be employed successfully in this context. In the present review, we hope to address this issue by discussing ongoing clinical trials in the use of gene therapy and gene editing technologies to treat genetic diseases, with a particular focus on specific challenges in the use of such approaches for treating mitochondrial diseases.

## A Brief Primer On Mitochondrial Genetics

**Fig. 1:**
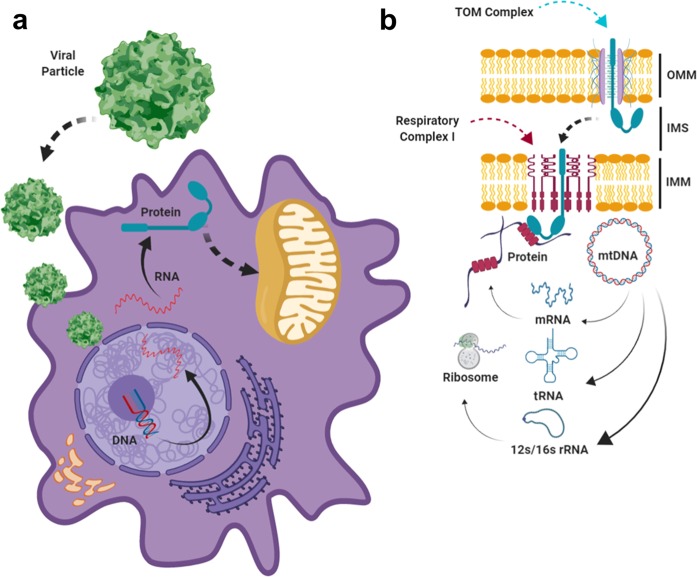
Expression of a putative nuclear-encoded mitochondrial protein using a recombinant viral vector. The majority of the proteins required for mitochondrial function (~1500) are encoded by the nuclear genome (nDNA), while a small subset of proteins (13), 22 tRNA and 2 rRNA are encoded by the mitochondrial genome (mtDNA). In the case of proteins encoded by the nuclear genome, restoration of protein function will involve transduction of the recombinant viral vector, transcription of the transgene, and translation of the protein in the cytosol (**a**), followed by transportation of the protein into the mitochondria through specialized import pathways (**b**). Most nuclear-encoded proteins are imported as precursors through the general “translocase of the outer membrane” (TOM) complex, which is located in the outer membrane. Subsequent import mechanisms differ based on the structure and function of the mitochondrial protein, as well as its ultimate destination. In the case of the example shown here, which is a protein destined for one of the respiratory complexes of the IMM, a “translocase of inner membrane” complex such as TIM23 (not shown) will interact with the TOM complex to facilitate insertion of the respiratory complex protein into the IMM. In contrast, mtDNA-encoded proteins are synthesized inside the matrix, and co-translationally inserted into the inner mitochondrial membrane to form complexes with their nDNA-encoded partners. By default, any proteins encoded by gene therapy vectors such as AAV will also be translated in cytosol like any other nDNA-encoded protein. Thus, in order to allotopically express an mtDNA-encoded protein from the nucleus, additional modification of a mtDNA-encoded protein will be required to make sure that it is imported to its proper location inside the mitochondria. Abbreviations: OMM (outer mitochondrial membrane), IMM (inner mitochondrial membrane), IMS (Intermembrane space), mtDNA (mitochondrial DNA), AAV (adeno-associated Virus).

The majority of the proteins required for mitochondrial function are encoded by the nuclear genome (nDNA), with over 1500 genes in nDNA estimated to be involved in mitochondrial structure and function. These genes are transcribed and translated outside of the mitochondria, and then transported into the mitochondria through specialized import pathways (see Fig. 1). Since mitochondria possess a two-layered lipid membrane—referred to as the outer (OMM) and inner (IMM) mitochondrial membranes—import pathways must use signal peptides to localize the proteins to their proper locations within the mitochondria, where they perform biochemically distinct functions. A series of protein complexes in the OMM and IMM cooperate to traffic each mitochondrial protein to its proper location.^6^ Most mitochondrial proteins encoded from the nDNA are imported through the OMM via the TOM (translocase of the outer membrane) complex, but a subset of proteins are imported via other means (e.g. β-barrel proteins, which are imported to the OMM via the Sorting and Assembly Machinery Complex). Subsequent localization to the intermembrane space, inner mitochondrial membrane, or matrix rely on distinct pathways such as the MIA (mitochondrial intermembrane space import and assembly) pathway or the TIM (translocase of the inner membrane) complexes. Proper trafficking and localization is crucial to the function of each of these nuclear-encoded mitochondrial proteins, and may present significant challenges if not accounted for in the gene replacement strategy being employed.

In contrast to the nuclear genome, the mitochondrial genome (also referred to as mitochondrial DNA, or mtDNA) is a circular DNA molecule about ~16.5 kilobases in length that is normally harbored in the mitochondrial matrix (inside the IMM) (Fig. 1b). The mtDNA encodes a small but critical subset of genes, including 13 protein-coding genes required for OXPHOS, as well as 22 tRNA and 2 rRNA genes required for the translation of those 13 protein-coding genes.^7–9^ The protein-coding genes are all components of respiratory complexes in the IMM that also contain proteins encoded by the nuclear genome. However, in contrast to the import pathways utilized to bring their nuclear-encoded counterparts to the mitochondria, mtDNA-encoded proteins are synthesized inside the matrix and co-translationally inserted directly into the IMM (Fig. 1b) by the mitochondrial ribosome, with the aid of the insertase Oxa1, the inner membrane protein Mba1, and a variety of other factors.^10–13^

Mitochondrial DNA is exclusively inherited from the mother. Therefore, a woman with mutant mtDNA can pass the disease directly through female offspring, resulting in heritable genetic afflictions that can be transmitted for multiple generations down the maternal line. In addition, most cells in the body contain hundreds of mitochondria that continually fuse and divide with each other to form a dynamic, filamentous network of organelles, with each individual mitochondrion carrying up to 10 copies of the mtDNA.^14^ When the mtDNA molecules within a cell are nearly identical at the nucleotide level, it is referred to as homoplasmy. In contrast, any situation resulting in divergent mtDNA sequences, whether from de novo mutations or variants passed down from an ancestor, is referred to as heteroplasmy.^15,16^ Several factors—such as the reactive oxygen species which arise as byproducts of OXPHOS, or the low fidelity of the mitochondria-specific DNA polymerase gamma—result in the accumulation of new and potentially deleterious mtDNA mutations with age.^17–19^ On top of this, significant changes in heteroplasmy levels for pre-existing mtDNA variants can occur over time as cell lines proliferate,^20^ and can even occur between mothers and their children.^21^ The latter phenomenon of heteroplasmy shift from between child and mother appears to be the result of a genetic “bottleneck” in the germline, wherein only a subset of mtDNA molecules replicate during a key phase of oocyte development.^22–24^ This can result in dramatic and unpredictable changes in heteroplasmy frequencies across generations. While many mtDNA variants may be benign or have limited effects on an individual, other variants can have devastating consequences for the health of the patient by disrupting mitochondrial function. Often, the severity of the effect depends on the percentage of the mutant variant present in the heteroplasmic individual, which is commonly referred to as the threshold effect.^25,26^ The threshold can also vary depending on the tissue or organ in question; energy-demanding organs such as the brain or the heart will generally have lower thresholds than less energy-intensive organs such as the kidney.^27^ As we will see below, all of these factors can present challenges to the approaches utilized in gene therapy to correct inheritable or somatic mutation of mtDNA.

## Advances In Sequencing Technologies Significantly Increase Molecular-Confirmed Patient Populations With Mitochondrial Disease

Methods for identifying disease-causing variants in the nuclear genome, such as whole genome or whole-exome sequencing, are a routine part of modern clinical practice. Likewise, identifications of pathological variants in mtDNA have become both straightforward and routine with the advent of next-generation sequencing. Currently, the gold-standard approach for sequencing mtDNA in patient blood samples is to selectively amplify the mtDNA by PCR and then utilize NGS for sequencing the resulting amplicons.^28–30^ Thus the patient’s haplogroup, the presence of any disease-causing variants, and the heteroplasmy level of said variants can all be determined in a single test.

While detection methods for mtDNA-mediated mitochondrial disease improves, prevention strategies for this group of diseases remain suboptimal. In the case of monogenic disorders transmitted through the nuclear genome, prenatal diagnosis and pre-implantation genetic diagnosis using embryo biopsies have proven valuable in preventing the transmission of genetic diseases from trait-carrying parents to their children. Such methods can, of course, also be employed just as effectively for the diagnosis of mitochondrial diseases carried in nuclear-encoded mitochondrial proteins. However, prenatal diagnosis for disease-causing mtDNA variants is a very different story. For a mother carrying a homoplasmic mutation, there is no need for prenatal diagnosis; all of her offspring are expected to be homoplasmic as well. However, for a woman carrying a heteroplasmic mutation, complications can often arise due to differences in heteroplasmy levels between the mother and offspring^31–33^ and differences in heteroplasmy levels across fetal tissues.^34^ Amniocentesis will be of limited use in such cases, as it mainly detects fetal skin cells and cells in fetal urine, making it difficult to predict the heteroplasmy levels in other tissues such as the brain. Even in the most optimistic of scenarios, prenatal diagnosis will only be able to prevent a small portion of pathogenic mtDNA transmission events, and mainly for those women with a low level of heteroplasmy. Since women with such a low level of heteroplasmy are often asymptomatic, many will continue to escape detection until they have already had one or more symptomatic offspring. Therefore, the ongoing improvement in sequencing technology suggests that more and more patients will continue to be identified for the foreseeable future. These new patients will need to be accurately diagnosed and treated alongside the already existing patient population even as treatment options lag behind, highlighting the importance of continued research into developing effective treatments for mitochondrial diseases.

## Special Considerations Based On Mitochondrial Biology

Based on the features of mitochondrial biology, several core issues must be tackled in any successful use of gene therapy in the treatment of mitochondrial disorders.

### Mitochondrial disorders often affect multiple organ systems and global expression of the transgene will be required

Previous gene therapy trials have largely focused on the treatment of conditions affecting specific tissues, such as optic^1^ or neurological disorders,^2,35,36^ which only require transgene delivery to very specific locations. Unfortunately, most mitochondrial disorders affect multiple organ systems, and will thus require the rescue vector to be expressed throughout the body in order to produce any significant improvement in the patient’s condition. This increases the risk of an immune response to the delivery vector, among other issues. It also requires practical consideration of how to achieve such a broad expression pattern. First, in order to even hope to achieve systemic effects, a large number of viral particles will need to be produced, which will increase the cost of treatment. Second, a viral particle with the correct tropism must be chosen based on the tissue that needs to be targeted. In most cases, systematic delivery across multiple tissues will be required. Furthermore, since the central nervous system is a particularly common target of mitochondrial diseases, the viral particle will also need to be able to cross the blood-brain barrier. A modified AAV serotype (AAV-PHP.B) has been recently developed through the use of a Cre-based targeted evolution strategy that has a remarkable tropism for CNS tissues in C57BL/6 mice, even when delivered intravenously.^37^ However, this particular serotype has been shown to lack such tropism following intravascular injection in other mouse strains,^38^ as well as in non-human primates.^39^ It has also been shown to cause acute toxicity issues in non-human primates when delivered at the kinds of high dosages that would be needed to restore mitochondrial function in patients.^38^ Both facts raise serious questions as to its utility in human patients. Despite these limitations of AAV-PHP.B itself, however, it is likely that even more refined delivery vectors will be developed in the coming years specifically tailored for use in human patients.

### Protein import into the mitochondria

Any mitochondrial protein encoded by nDNA must be imported into the mitochondria through a series of complex import channel proteins (Fig. 1). Since the mitochondrial proteins encoded by the mtDNA do not normally contain such signals (since they are synthesize within the mitochondria to begin with) (Fig. 1), a way must be found to import them into the mitochondria when they are expressed from a viral vector. This can be most easily accomplished by adding a mitochondrial targeting signal (MTS) to the mtDNA-encoded protein, effectively leveraging the natural import system to import the protein to its correct location in the mitochondrion. For the mtDNA-encoded RNAs (22 tRNA and two rRNA), it is considerably more challenging to effect their delivery to the mitochondrial matrix. While the import of small non-coding RNAs has been observed throughout eukaryotes, the process is not nearly as well-understood as the mitochondrial protein import pathway, nor does it appear to be as efficient.^40^ However, there have been promising preliminary results in this area, including the discovery of a 20-bp RNA sequence that appears to facilitate the import of both non-coding RNAs as well as mRNAs.^41^

Unfortunately, for these mtDNA-encoded proteins, even forcing the import of an allotopically-expressed version of the protein may not be as simple of a solution as it initially appears. It has been recently demonstrated that the overproduction of mitochondrial proteins (whether encoded by mtDNA or nDNA) may, in and of itself, cause severe defects in mitochondrial function and metabolism. Production of defective and/or misfolded mitochondrial proteins encoded from the nuclear genome can lead to a toxic buildup of mitochondrial protein precursors in the cytosol (a process referred to as mitochondrial precursor overaccumulation stress, or mPOS), as well as dysfunction within the mitochondria itself (including disrupted OXPHOS, proteotoxic stress, and mtDNA depletion) due to the accumulation of misfolded proteins.^42^ More importantly, there are even indications that overexpression of an otherwise wild type, nuclear-encoded mitochondrial protein can trigger mPOS in human cells through over-crowding.^43^ If true, this would represent a major challenge to the use of gene therapy to replace defective mitochondrial proteins encoded from either genome, as high expression levels are generally required to produce any significant improvements in a patient’s condition.

### Issues related to unbalanced gene expression

Even assuming that an overexpressed, nDNA-encoded mitochondrial protein can be imported normally, there remains a possibility that excess protein will interfere with mitochondrial function, as components of the electron transport chain must be present in precisely balanced ratios in order for efficient OXPHOS to occur. If one component is over- or underrepresented in the electron transport chain, reactive intermediates can build up and the levels of intact complexes may be reduced. The ultimate result will be an overproduction of reactive oxygen species that may damage the cell in the long term. Given that different tissues can have different levels of heteroplasmy, such a situation has the potential to become truly complex: normal tissues may end up expressing excessive amounts of the mitochondrial protein in question even as the phenotype is “rescued” in diseased tissues.

## Gene Therapy In Relation To Primary Mitochondrial Disorders

Gene replacement therapy based around the AAV2 vector has shown modest, but promising, success in mice for a variety of nuclear-encoded mitochondrial disease genes.^44–46^ However, the only primary mitochondrial disease currently involved in active clinical trials of gene replacement therapy is Leber’s hereditary optic neuropathy (LHON), caused by mutations in the mitochondrially-encoded *MT-ND4* gene. Multiple trials are currently investigating the treatment of LHON using AAV2 vectors and *MT-ND4* coding sequences modified to carry an MTS. In general, these trials have produced some improvements in visual function for patients suffering from LHON, particularly in those with a disease course shorter than 2 years prior to the time of treatment.^47^ However, it has been noted in at least one of the trials (NCT02652780) that injection of the viral vector into one eye also restored function in the uninjected eye. A qPCR-based analysis appeared to explain this result by showing that the viral vector could be detected in tissues extract from both the injected as well as the uninjected eye. This may suggest that viral vector was transferred from the injected to the uninjected eye via the optic nerves at the optic chiasm, although this mechanism has yet to be fully confirmed. Overall, however, the results of these trials have been generally encouraging, and there is reason to believe that other mitochondrial disease genes may be similarly amenable to gene replacement therapy, so long as protein import and expression level issues are properly addressed. Thus far, very few clinical trials have been conducted with nDNA-related mitochondrial diseases.

### Mitochondrial Replacement Therapy (MRT)

In light of the difficulties involved in applying traditional gene therapy to the treatment of pathogenic mtDNA variants, mitochondrial replacement therapy (MRT) has generally been regarded as the most effective technique available for the prevention of inherited mtDNA mutations.^48–50^ In this approach, the nuclear genome from a mother carrying a deleterious mtDNA mutation is physically transferred, through micromanipulation techniques, into an enucleated oocyte from another healthy female with no mtDNA mutations. There is actually a broader interest in this technique in the field of reproductive medicine, as age-related decline in mitochondrial function is considered to be a major contributor to the decline in oocyte quality and fertility that occurs in women over the age of 35.^51,52^ For this reason, there is a great deal of interest in utilizing MRT to allow older woman to transfer their nuclear genome to oocytes from younger woman and thus conceive genetically-related offspring. However, there is some debate as to the ethical justification for using such an extreme intervention for relatively mundane fertility issues. In contrast, the ethical case for using MRT to prevent the transmission of serious mtDNA mutations is much more straightforward.

There are several approaches currently employed for MRT, including polar body nuclear transfer,^53,54^ pronuclear transfer,^55,56^ and spindle-chromosome complex transfer.^57,58^ Each approach differs in the timing of the transfer (i.e., before or after fertilization) as well as the composition of the material transferred, and there remains a lively debate in the field as to the relative merits of each approach. However, no matter the specific approach employed, they all result in offspring that is genetically related to the patient (mother) and father at the level of the nuclear genome, but who will carry mtDNA from the oocyte donor and thus suffer none of the health consequences of the patient’s mtDNA mutation.^59^

Beyond the novel ethical issues raised by the notion of this kind of “three-parent” baby, there is a practical safety concern with MRT regarding the amount of mutated mtDNA carried over from the patient during the process of transferring the nuclear material. No matter how precisely done, there is always some cytoplasmic material carried over when the nuclear material is extracted and transferred. So long as appropriate care is taken to minimize the amount of cytoplasmic carry-over (in the most optimized methods, less than 2%),^59^ the resulting heteroplasmy appears to be quite low.^60^ This is also borne out by reports from individuals who were born as a result of ooplasmic transplantation (an earlier and less sophisticated alternative to MRT), who appear to be largely normal in terms of health and cognitive abilities.^61,62^ There remains a possibility that the mutant mtDNA frequency may drift upwards as the child grows older, or in later generations.^60^ However, careful planning and selection of compatible donor haplogroups can do a great deal to mitigate the former, and the selection of only male embryos for implantation effectively eliminates the latter risk. Certainly, the case of the first human child produced by MRT in 2016 is encouraging,^63^ as said child remains free of any health issues as well as any significant shift in heteroplasmy level as of this writing.

### Difficulties in utilizing CRISPR-based gene editing for mtDNA

MRT offers a powerful but restricted approach. It can only be used to preemptively stop the transmission of pathological mtDNA variants, and does nothing to help existing patients. It is incumbent upon us to consider ways to incorporate more effective genome-editing techniques into their therapy.

The most obvious approach would be to utilize the highly-celebrated CRISPR gene editing technique. However, this may not end up being the most effective approach, as it requires two components in order to introduce double-stranded breaks into the genome: the Cas9 nuclease for cutting the DNA, and a guide RNA (gRNA) that determines the DNA sequence that is to be targeted. In addition, if specific alterations are to be made to the mtDNA sequence, a homologous repair template must be present alongside the Cas9 protein and gRNA. Since mtDNA is located inside mitochondria, all three components must be imported into the mitochondria in order for editing to occur. This is further complicated in the oocyte and zygote, which are estimated to contain over 100,000 mitochondria. This means that the process of importing various CRISPR components, as well as enzyme efficiency in editing the mutant mtDNA molecules themselves, must be extraordinarily efficient in order to have an impact on the heteroplasmy level of the oocyte or newly fertilized zygote. Together, these factors create an additional set of logistical complications that are not present in CRISPR-based editing of nuclear genes.

One report from 2015 claimed that the CRISPR/Cas9 system can be used to selectively edit the mtDNA in cultured human cells,^64^ but the mechanisms that would allow such a process to occur are somewhat unclear. It appears that the Cas9 protein, which was already modified to carry an MTS, binds to gRNA in the cytoplasm and helps to transport it into the mitochondria, neatly solving the import problem for gRNA. Gammage and colleagues have extensively discussed this paper and other related issues in their 2018 review of the topic, and conclude that the evidence supporting CRISPR/Cas9 editing of mtDNA remains ambiguous.^65^

A very recent paper in zebrafish also appears to show that a single-stranded DNA targeting cassette containing homolog arms specific to the mitochondria genome was able to generate homologous recombination events when combined with the CRISPR/Cas9 system. This would be a truly game-changing finding if true, as it would allow any desired nucleotide change to be induced in the mtDNA, rather than simply manipulating the heteroplasmy levels of the existing mtDNA populations through selective degradation. To help explain this surprising result, the authors present data showing that the CRISPR/Cas9 system appears to significantly upregulate several of the major proteins involved in nuclear DNA repair.^66^ Once again, however, the precise mechanism remains unclear. Most critically, it has not been clearly demonstrated that the nuclear DNA repair enzymes are even imported into the mitochondria, nor that they can effectively assemble and operate properly within the mitochondrial matrix, which would be an obvious mechanistic prerequisite for any homologous recombination events to be able to occur. Certainly there is little evidence that homologous recombination of mtDNA occurs with any significant frequency in mammals,^67^ even under the harsh conditions that would be expected to select for such events.^68,69^ Furthermore, when recombination events do occur, they appear to be overwhelmingly intramolecular in nature,^70^ with only rare instances of intermolecular exchange of DNA sequences between mtDNA molecules. Thus, independent verification will be necessary to determine the accuracy of this claim. However, even assuming that the homologous recombination machinery can operate effectively within the mitochondrial matrix, the overexpression of homologous repair and DNA repair enzymes can lead to genome instability^71–73^ which could end up significantly harming the patient. Thus, if CRISPR/Cas9 does indeed cause increased expression of DNA repair enzymes as the authors claim, then this would need to be accounted for and mitigated against in all future implementations of CRISPR/Cas9-based genome repair.

Given the fraught nature of mtDNA editing based on homologous repair, the most popular and effective option for editing mtDNA at this moment is to utilize restriction endonucleases that selectively cut mutant mtDNA molecules while leaving wild type mtDNA molecules intact. The potential of this approach lies in the proofreading exonuclease activity of mitochondrial polymerase gamma, which has been shown to aggressively eliminate linearized mtDNA molecules as part of its intrinsic activity.^74,75^ Under normal circumstances, this exonuclease activity appears to reduce the formation of mtDNA deletions, whose frequency increases when linear mtDNA molecules persist.^75^ In the context of endonuclease based mtDNA editing, this allows for the efficient and selective elimination of mutant mtDNA independent of homologous repair mechanisms. Once the mutant mtDNA has been eliminated in this way, the wild type mtDNA is then free to repopulate the mitochondria in the cell to homoplasmy or near homoplasmy. Approaches using nucleases that do not require gRNA, such as TALENs^76^ and ZFNs,^77^ have been successful in mice, and are so far the only proven means of altering mtDNA heteroplasmy in the lab. For this reason, they are also the most likely option for clinical use for the foreseeable future.

## Conclusions

Recent successes in the field of gene therapy are truly encouraging and are likely only a glimpse of the progress to come in the near future. It is our belief that these cutting-edge genetic techniques can also significantly improve the lives of many of the patients and families who currently suffer under the burden of mitochondrial disease. However, caution must be taken to properly account for the unique qualities of the mitochondrial organelle in order to fully realize the potential of this technology in the treatment of mitochondrial disorders. For example, the current literature clearly demonstrates that mtDNA editing via protein-only nucleases such as TALENs or ZFNs is a much more effective approach than CRISPR/Cas9-based editing, and that the former approach must be prioritized for any near-term clinical trials. Furthermore, the delivery approach must take into account the relevant properties of each mitochondrial protein in question, in particular their localization within the mitochondrial organelle and how they will be properly targeted to that location without overwhelming the mitochondrial import machinery. The preliminary success enjoyed by the recently published clinical trials suggest that these challenges, while significant, are far from insurmountable.

## Data Availability

There is no data associated with this paper.
